# FOXN3 controls liver glucose metabolism by regulating gluconeogenic substrate selection

**DOI:** 10.14814/phy2.14238

**Published:** 2019-09-25

**Authors:** Santhosh Karanth, Bhagirath Chaurasia, Faith M. Bowman, Trevor S. Tippetts, William L. Holland, Scott A. Summers, Amnon Schlegel

**Affiliations:** ^1^ University of Utah Molecular Medicine Program Salt Lake City Utah; ^2^ University of Utah Diabetes and Metabolism Research Center Salt Lake City Utah; ^3^ Department of Nutrition and Integrative Physiology College of Health University of Utah Salt Lake City Utah; ^4^ Department of Biochemistry University of Utah School of Medicine Salt Lake City Utah; ^5^ Division of Endocrinology, Metabolism and Diabetes Department of Internal Medicine University of Utah School of Medicine Salt Lake City Utah

**Keywords:** FOXN3, glucose, glutamine, liver, mouse, MYC, pyruvate

## Abstract

The *FOXN3* gene locus is associated with fasting blood glucose levels in non‐diabetic human population genetic studies. The blood glucose‐modifying variation within this gene regulates the abundance of both FOXN3 protein and transcript in primary human hepatocytes, with the hyperglycemia risk allele causing increases in both FOXN3 protein and transcript. Using transgenic and knock‐out zebrafish models, we showed previously that FOXN3 is a transcriptional repressor that regulates fasting blood glucose by altering liver gene expression of MYC, a  master transcriptional regulator of glucose utilization, and by modulating pancreatic α cell mass and function through an unknown mechanism. Since homozygous *Foxn3* null mice die perinatally, and heterozygous carries of the null allele are smaller than wild‐type siblings, we examine the metabolic effects of decreasing mouse liver Foxn3 expression in adult life, performing dynamic endocrine tests not feasible in adult zebrafish. Fasting glucose, glucagon, and insulin; and dynamic responses to glucose, insulin, pyruvate, glutamine, and glucagon were measured. Gluconeogenic and amino acid catabolic gene expression was examined in livers, as well. Knocking down liver *Foxn3* expression via transduction with adeno‐associated virus serotype 8 particles encoding a short hairpin RNA targeting *Fonx3* decreases fasting glucose and increases *Myc* expression, without altering fasting glucagon or fasting insulin. Liver Foxn3 knock‐down confers increases glucose tolerance, has no effect on insulin tolerance or response to glucagon challenge, blunts pyruvate and glutamine tolerance, and modulates expression of amino acid transporters and catabolic enzymes. We conclude that liver Foxn3 regulates substrate selection for gluconeogenesis.

## Introduction

The transition from fed to fasted states is marked by simultaneous hormonal, neuronal, and intrinsic nutrient sensing changes within the liver. These signals culminate in altered intermediary metabolism to generate glucose and ketone bodies for export for use by other organs (Newman and Verdin, 2017). The genetic networks controlling these signaling and metabolic pathways are subjects of intense investigation, since elucidating the response to nutrient deficiency holds diagnostic and therapeutic promise for type 2 diabetes mellitus, obesity, non‐alcoholic fatty liver disease, and related illnesses.

A single nucleotide polymorphism within the first intron of the human *FOXN3* gene is independently and statistically significantly associated with fasting blood glucose (Manning et al., 2012). The encoded Forkhead superfamily transcriptional repressor had not been implicated in metabolic regulation previously, providing an opportunity to reveal new aspects of fasting metabolism. The hyperglycemia risk allele of this variation drives increased expression of liver FOXN3 (Karanth et al., 2016). We modeled this increase in liver FOXN3 by transgenic over‐expression of human *FOXN3* in zebrafish hepatocytes, and observed an increase in fasting blood glucose in animals fed their normal diets (Karanth et al., 2016). Conversely, deletion of the *foxn3* ortholog in zebrafish decreases fasting glucose (Karanth et al., 2018). On a mechanistic level, FOXN3 regulates fasting blood glucose by directly repressing expression of MYC (Karanth et al., 2016), a transcription factor central to promoting glucose utilization in liver (Sloan and Ayer, 2010). Quite surprisingly, liver FOXN3 gene dose regulates pancreatic islet α cell biology, modulating both plasma glucagon and α cell abundance in zebrafish, with the liver‐transgenic model showing increased α cell mass and the deletion model showing decreased α cell mass (Karanth et al., 2018). Paralleling these zebrafish model studies, non‐diabetic human carriers of the hyperglycemia risk allele within the FOXN3 locus show blunted suppression of glucagon during an oral glucose tolerance test, while showing no differences in fasting glucagon; except for fasting glucose, these FOXN3 hyperglycemia risk allele carriers do not have different glucose or insulin parameters during the oral glucose challenge (Karanth et al., 2018). Further underscoring that liver FOXN3 does not appear to impact insulin action, carriers of the rs8004664 risk allele do not have altered glucose disposal rates when subjected to high‐dose euglycemic, hyperinsulinemic clamp (Erickson et al., 2019).

Since FOXN3 is required for craniofacial development (Schuff et al., 2007; Samaan et al., 2010), a viable loss‐of‐function mouse model to explore the role of this factor in adult liver metabolism is not available. Here, we use liver‐tropic adeno‐associated virus serotype 8 (AAV8) to knock‐down mouse *Foxn3* expression and examined the consequences on fasting glucose, glucagon, and insulin; and on the responses to intraperitoneal glucose, insulin, pyruvate, glutamine, and glucagon. We also measured select transcripts encoding proteins involved in amino acid transport and metabolism. Our studies reveal a role for FOXN3 in regulating gluconeogenic substrate selection.

## Materials and Methods

### Animals

All mouse studies in the Schlegel, Summers, and Holland laboratories were approved by the institutional animal care and use committee. Wildtype C57Bl/6J male mice were purchased from Jackson Laboratories (000664). Mice were maintained on Harlan Scientific 2920i diet.

**Figure 1:**
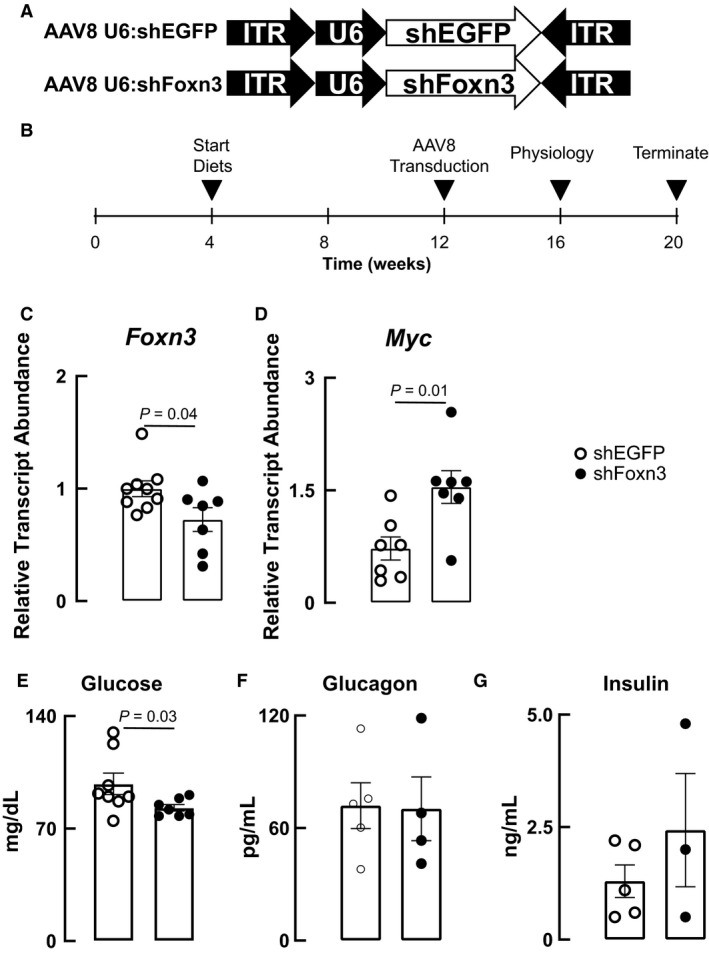
Knock‐down of *Foxn3* in mouse liver de‐represses *Myc* and lowers fasting glucose. (A) Viral constructs used to knock‐down EGFP or Foxn3 selectively in mouse liver. AAV8 particles were prepared using the strong RNA polymerase III promoter U6 to drive the shRNAs targeting *EGFP* and *Foxn3*; ITR, inverted tandem repeats. (B) Experimental design of the study. Mice were fed defined diets from 4 to 12 weeks of age, transduced with viruses, maintained on diets for an additional 4 weeks, and then subjected to analyses for fasting glucose, glucagon, insulin; glucose, insulin, pyruvate, and glutamine tolerance; and glucagon responses; and then sacrificed for gene expression analyses. (C and D) Transduction of AAV8 U6:shFoxn3 decreases liver Foxn3 and increases liver Myc transcripts. (E‐G) Knock‐down of Foxn3 decreases fasting blood glucose, but does not alter plasma glucagon or insulin after a 16‐h fast.

AAV8 particles bearing the U6 promoter and shRNAs directed against *EGFP* or mouse *Foxn3* were purchased from Vector Biolabs. Twelve‐week‐old mice were infected with 3 × 10^11^ genome copies per animal by retro‐orbital injection under anesthesia. The constructs used and the timeline of experimental procedures is presented in Figure 1A and B.

### Glucose, glugagon, and insulin measurements

Blood glucose was measured using a Bayer ContourNext EZ glucometer. Plasma glucagon and insulin were measured using a fluorescence resonance energy transfer‐based immunoassay from CISBio following the manufacturer's instructions (Gray et al., 2015; McCommis et al., 2015; Karanth et al., 2018).

### Dynamic endocrine tests

Starting at 16 weeks of age (4 weeks after transduction with AAV8 particles) and continuing to 20 weeks, mice were subjected to glucose tolerance, pyruvate tolerance, insulin tolerance, and glucagon challenge (in this order). Following each intraperitoneal injection, animals were returned to normal housing and feeding. Animals were allowed to recover for 1 week between intraperitoneal injections, with all dynamic endocrine tests concluding at 20 weeks of age. Prior to all intraperitoneal injections, mice were placed into fresh cages and fasted for the indicated amount of time. Hormones and metabolites were prepared in 0.9% saline for intraperitoneal injection. For glucose tolerance testing, animals were fasted for 12 h, and then injected with 2 g/kg glucose intraperitoneally. For pyruvate tolerance testing, animals were fasted for 12 h, and then injected with 2 g/kg pyruvate intraperitoneally. For glutamine tolerance testing, animals were fasted for 14 h, and then injected with 2 g/kg glutamine intraperitoneally (Gray et al., 2015; McCommis et al., 2015). For insulin tolerance testing, animals were fasted for 4 h, and then injected with 0.5 U/kg human insulin (Humulin R, Eli Lilly) intraperitoneally. For glucagon challenge, animals were fasted for 4 h, and then injected with 1 mg/kg human glucagon (Novo Nordisk) intraperitoneally. Blood was collected at the indicated time points following injection (between 0 and 120 min). At the conclusion of each test, animals were fed and returned to normal housing.

### Transcript quantification

At 20 weeks of age, over‐night‐fasted mice were euthanized by isoflurane inhalation and cervical dislocation. Livers were harvested and flash frozen in liquid nitrogen. Total RNA was extracted using a standard TRIzol method (Thermo Fisher). mRNA, from 2 μg of total RNA was converted to cDNA using oligo(dT) primer and random hexamers according to the manufacturer's instructions (Clontech EcoDry Premix), exactly as we described previously (Karanth et al., 2016). Transcripts for mouse *Foxn3* and *Myc* were quantified with RT‐PCR (Karanth et al., 2018). TaqMan probes and primer pairs for other transcripts measured in this study are listed in Table S1.

### Statistical analysis

GraphPad Prism 8 was used for two‐sided Student *t*‐test and curve integration. Results for individual mice are presented in scatter plots, with mean ± SEM bars overlaid (Weissgerber et al., 2015). For glucose, insulin, pyruvate, and glutamine tolerance test, results are plotted as graphs where each point is the mean ± SEM value; area under the curve was calculated by the software for individual animals, and the integrated values of these individual curves, normalized to control, were plotted as scatter plots with mean ± SEM bars overlaid.

## Results

### Liver‐limited knock‐down of *Foxn3* increases *Myc* expression, and decreases fasting blood glucose, without altering fasting glucagon or fasting insulin in mice

To examine the consequences of decreasing liver Foxn3 expression in adult mice, we prepared AAV8 encoding shRNAs targeting mouse *Foxn3* and *EGFP* (Fig. 1A). These shRNA were placed under the control of the U6 promoter to maximize RNA polymerase III‐dependent expression in liver (Grimm et al., 2006). Male C57Bl/6J mice were fed normal fat diets from 4 weeks of age to 12 weeks of age, infected with AAV8 U6:shEGFP and AAV8 U6:shFoxn3 particles (hereafter shEGFP and shFoxn3, respectively), and then subjected to a series of physiological studies (Fig. 1B). This experimental design parallels the normal diets fed to the zebrafish models we studied previously (Karanth et al., 2016, 2018). At the conclusion of the experiments, animals were sacrificed and livers were dissected and analyzed for *Foxn3*, *Myc* and select transcripts encoding gluconeogenic and amino acid catabolic proteins.

Transduction of shFoxn3 decreased *Foxn3* transcript by 40%, and increased the abundance of the direct Foxn3‐repressional target *Myc* transcript (Karanth et al., 2016) by 1.5‐fold compared to transduction of shEGFP (Fig. 1C and D). These results mirror the effects seen in zebrafish mutants carrying a heterozygous *foxn3* loss‐of‐function allele where a 50% reduction in *foxn3* transcript was accompanied by a twofold increase in *mycb* transcript (Karanth et al., 2018). These effects are also similar to that observed when immortalized human cells are transduced with a dominant‐negative FOXN3 cDNA that occurs in leukemia as a consequence of pathological intron polyadenylation (Lee et al., 2018). Knock‐down of *Foxn3* in mouse livers decreased fasting blood glucose (Fig. 1E). Fasting blood glucagon and insulin were unaffected by shFoxn3 (Fig. 1F and G). Collectively, these results indicate that shFoxn3 transduction in mouse liver recapitulates the effects seen in livers of zebrafish with life‐long heterozygous deficiency of the encoding gene when fed normal fat diets. Critically, mice transduced with shFOXN3 had the same mass and body composition as diet‐matched shEGFP transduced mice throughout the study (not shown).

### Liver‐limited knock‐down of *Foxn3* enhances glucose tolerance, has no effect on insulin tolerance, causes intolerance to pyruvate and glutamine, and does not alter glucagon stimulation in mice

**Figure 2:**
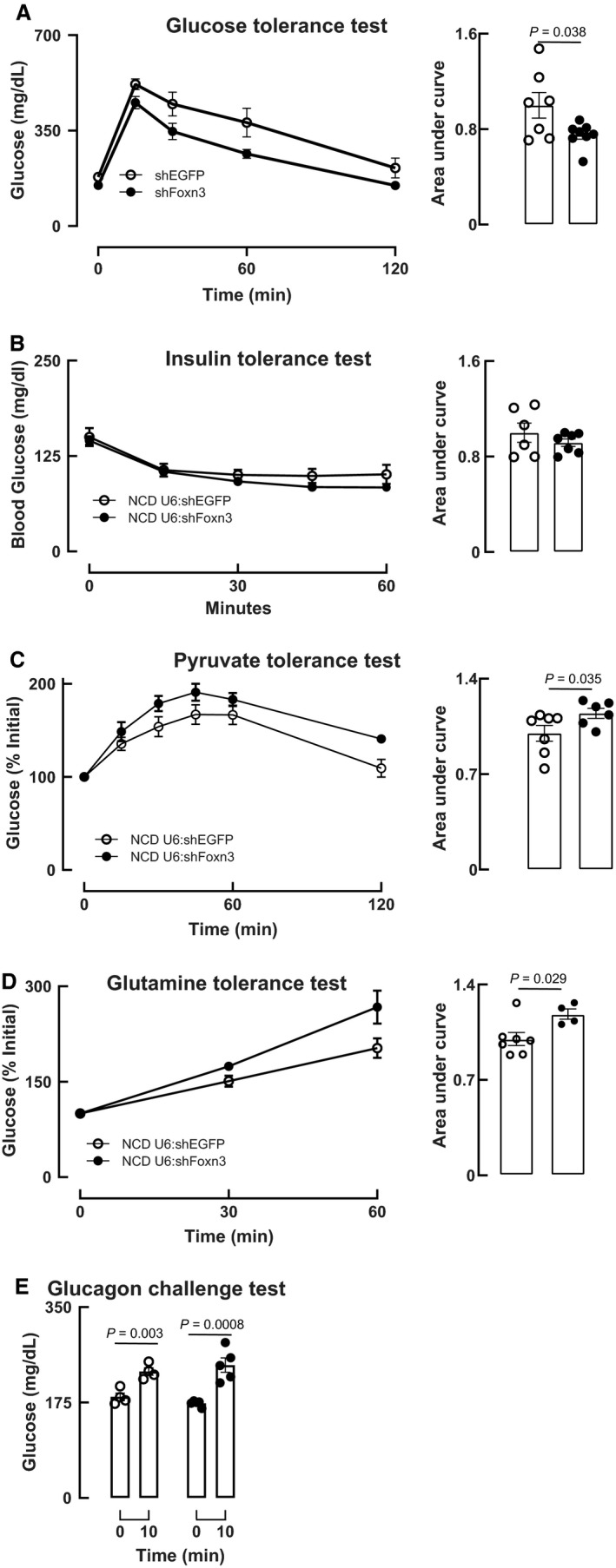
Dynamic responses to glucose, insulin, pyruvate, glutamine, and glucagon when liver *Foxn3* is knocked down. Knock‐down of *Foxn3* (shFoxn3) improves glucose tolerance (A), does not alter insulin tolerance (B), causes pyruvate and glutamine intolerance (C and D), and does not alter glucagon‐stimulated glucose increases in mice (E).

To explore the effects of knocking‐down liver *Foxn3* on metabolic and hormonal responses, we performed a series of dynamic endocrine tests. Knock‐down of *Foxn3* in liver enhanced intraperitoneal glucose tolerance, as reflected by a lower peak blood glucose and lower area under the curve (Fig. 2A). *Foxn3* knock‐down in liver did not alter intraperitoneal insulin tolerance (Fig. 2B).

We previously found that zebrafish carrying one null *foxn3* allele had decreased gluconeogenic gene expression (Karanth et al., 2018). To gauge the impact of liver‐limited Foxn3 knock‐down on gluconeogenic substrate handling, we performed pyruvate and glutamine tolerance tests. Unexpectedly, Foxn3 knock‐down led to intolerance to both pyruvate and glutamine (Fig. 2C and D). Finally, when challenged with glucagon, shFoxn3‐transduced mice showed unchanged blood glucose response compared to shEGFP‐transduced mice (Fig. 2E).

### Liver‐limited knock‐down of *Foxn3* causes changes in amino acid catabolic gene expression in mice

**Figure 3:**
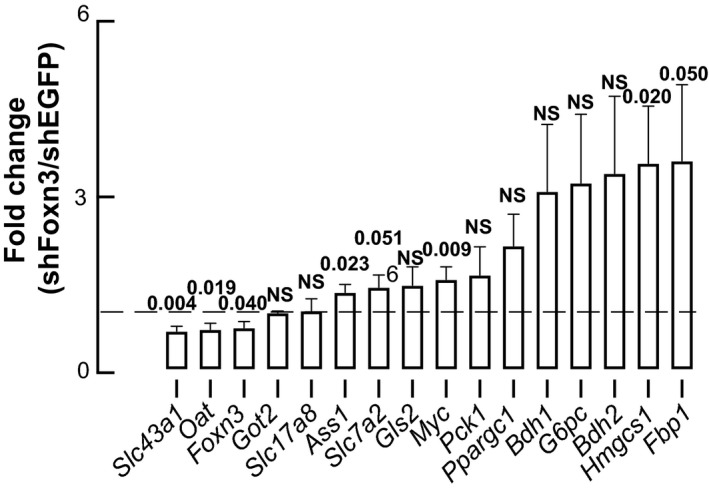
Changes in select mRNAs expressed in liver when Foxn3 is knocked down. Results are presented as fold‐change ± SEM relative to shEGFP (line of unity), arranged from lowest to highest, with *P* values shown, *n* = 7–9 per cohort. Actb was used to normalize RNA abundance.

Next, we performed a targeted survey of transcripts encoding proteins involved in amino acid transport and catabolism (*Ass1*, *Gls2*, *Got2*, *Oat*, *Slc7a2*, *Slc17a8*, *Slc43a1*) ketogenesis(*Bdh1* and *Hmgcs2*), gluconeogenesis (*Fbp1*, *G6pc*, *Fbp1*), and transcriptional regulation  (*Ppargc1*, Fig. 3). Many of these genes are regulated by glucagon (Watanabe et al., 2012; Longuet et al., 2013; Solloway et al., 2015; Dean et al., 2017; Kim et al., 2017), which alters amino acid metabolism to enhance gluconeogenesis. Knock‐down of *Foxn3* significantly decreased expression of *Slc43a1*, *Oat*, and *Ass1*, and increased expression of *Hmgcs1 *and *Fbp1*. As internal controls, *Foxn3* and *Myc* transcripts were measured, and were significantly decreased and increased, respectively, confirming persistence of the AAV8 infection and the encoded shRNAs' effects.

## Discussion

Here, we prepared and characterized an adult mouse model of liver‐limited *Foxn3* knock‐down. We conducted fasting and dynamic endocrine tests on adult mice transduced with liver‐tropic AAV8 viruses carrying shRNA constructs that decrease *Foxn3* gene expression in order to probe the mechanism through which this transcriptional repressor regulates fasting glucose metabolism and substrate handling. Confirming our previous findings in life‐long, global, zebrafish mutants, knock‐down of *Foxn3* in adult mouse liver decreased fasting blood glucose and de‐repressed expression of Myc. In this study, decreased *Foxn3* expression in adult mouse liver improves glucose tolerance, without modulating insulin tolerance. Surprisingly, knock‐down of *Foxn3* led to pyruvate and glutamate intolerance. Similarly, *Foxn3* knock‐down altered expression of select amino acid transporters (decreased Slc43a1) and enzymes (decreased Oat, Got2, Ass1; and increased Bdh2 and Hmgcs1). These changes in gene expression reveal a broader role for *Foxn3* in regulating intermediary metabolism than our previous, glucose‐focused studies predicted. A comprehensive gene survey in mice where liver *Foxn3* is knocked out is likely to extend these findings, and allow for more thorough physiological analyses.

Since glucagon specifically directs glutamine‐derived carbon atoms to gluconeogenic flux (Miller et al., 2018), our findings suggest that *Foxn3* modulates gluconeogenesis by influencing expression of metabolic enzymes and transporters that impact substrate selection. Future studies with stable isotope tracer approaches will definitely address this issue. Likewise, the role of liver Foxn3 in feeding back to the endocrine pancreas (regulating α cell mass) merits further exploration (Karanth et al., 2018). The interaction among liver *Foxn3* gene dose and diet (e.g., high fat) and additional genetic factors will also be investigated.

Because 80% chromatin sites occupied by FOXN3 in HepG2 cells lack both the canonical Forkhead DNA sequence and an alternative (Forkhead‐like) DNA sequence (Nakagawa et al., 2013, Rogers et al., 2019), identifying direct and indirect Foxn3 target genes impacting metabolism will require parallel genomic and non‐genomic efforts. Specifically, comprehensive gene expression, fluxomic, and physiological methods (e.g., euglycemic hyperinsulinemic clamp) will reveal precisely how Foxn3 regulates liver intermediary metabolism. Conversely, the mechanism(s) through which glucagon causes rapid degradation of Foxn3 protein, and decreases Foxn3 transcript abundance (Karanth et al., 2016, 2018) merit consideration in future studies. The latter mode of regulation may reveal a role for glucagon in directing pre‐mRNA intron polyadenylation (Lee et al., 2018), pre‐mRNA degradation (Fish et al., 2019), or suppression of mRNA translation by several microRNAs (Kong et al., 2019), all mechanisms used by cancers to selectively silence Foxn3 expression. Finally, our overall hypothesis that Foxn3 alters gene expression programs regulating glucagon responsiveness does not exclude the possibility that liver Foxn3 alters the clearance of glucose or amino acid metabolism by other organs.

## Conflict of Interest

The authors declare no conflict of interest.

## Supporting information




**Table S1.** Primer pairs and TaqMan probes for the transcripts examine in this study.
