# Supplementary material for: FOXN3 controls liver glucose metabolism by regulating gluconeogenic substrate selection
Source: Physiol Rep. 2019 Sep 25;7(18):e14238. doi: 10.14814/phy2.14238 (PMC6759504; doi:10.14814/phy2.14238)
Supplement: Supplementary file 1 — Table S1. Primer pairs and TaqMan probes for the transcripts examine in this study. [file PHY2-7-e14238-s001.docx]

Table S1. Primer pairs and TaqMan probes for the transcripts examine in this study.

**Primer Pairs**

| **Gene** | **Forward primer** | | | **Reverse primer** | |  |
| --- | --- | --- | --- | --- | --- | --- |
| ***Slc17a8*** | CCATACCAAAGGAGTGGCTATC | | | GTCCAGGTGGTTGACATTGA | |  |
| ***Slc43a1*** | CTCTCATCAGTGCCGTGTTT | | | TCCCAGGAACGAGAGAAGTAG | |  |
| ***Slc7a2*** | CCGGGATGGCTTACTGTTTAG | | | AAGGCCATCACAGCAGAAA | |  |
| ***Oat*** | GAGAGGGAAAGGGTTGCTAAA | | | GAAGCCCGTTATCTCGAAGTC | |  |
| ***Ass1*** | GGAATGAAGTCCCGAGGTATC | | | CGTGAAGGCCTCTATGTCTAAA | |  |
| ***Got2*** | AGCAACCATCCTGACTTCTC | | | GAGCCCTCTTTCTTCAGGTT | |  |
| ***Actb*** | CTCTGGCTCCTAGCACCATGAAGA | | | GTAAAACGCAGCTCAGTAACAGTCCG | |  |
|  | |  |  | |  | |
| **TaqMan Probes** | |  |  | |  | |
| **Gene** | **Thermofisher catalog #** | | |  |  |  |
| ***Foxn3*** | Mm02343199_g1 | | |  |  |  |
| ***Myc*** | Mm0048704_m1 | | |  |  |  |
| ***Actb*** | Mm01205647_g1 | | |  |  |  |
| ***Ppargc1*** | Mm01208835_m1 | | |  |  |  |
| ***Bdh1*** | Mm00558330_m1 | | |  |  |  |
| ***Gls2*** | Mm01164862_m1 | | |  |  |  |
| ***Pck1*** | Mm01247058_m1 | | |  |  |  |
| ***Bdh2*** | Mm00459075_m1 | | |  |  |  |
| ***G6pc*** | Mm00839363_m1 | | |  |  |  |
| ***Hmgcs1*** | Mm01304569_m1 | | |  |  |  |
| ***Fbp1*** | Mm00490181_m1’ | | |  |  |  |
